# Mexican Sanitation; Sanitation on the Rio Grande

**Published:** 1893-11

**Authors:** 


Mexican Sanitation.—There has been much complaint that our sister republic does not enforce vaccination, or take other steps to prevent the spread of small-pox in that country, and the consequence is a perpetual danger of its invading Texas. The Rio Grande, which for a great length marks the boundary line, is shallow and fordable at so many places that it would require an army of men to successfully guard against invasion of infection. This matter was the subject of a paper by our State Health Officer, Dr. Swearingen, read at the Mexico meeting of the American Public Health Association in November, 1892, and in a paper on Texas Quarantine, read by him at the recent Public Health Congress in Chicago, he again entered a protest. Some of the Mexican delegates, who fully appreciated the situation, stated that they have good sanitary laws in Mexico, but cannot enforce them. They have separate state governments, as we have, and a federal government.

The Journal is pleased to be able to announce that the protests of our State Health Officer are already bearing good fruits, and an important movement by the Mexican federal government toward co-operation with Texas has been inaugurated on the Rio Grande, at least at one point.

Dr. T. J. Turpin, the efficient Texas State Quarantine Officer at Laredo, writes to State Health Officer Swearingen:

"I am informed by Don Pedro Arguelles, collector of customs, New Laredo, Mexico, that a plant for disinfecting has just been received there, and that it will be erected within the next two weeks. The plant consists of an iron room, with furnace and engine for heating steam, trucks for running the baggage in, etc. There will also be a room for bathing passengers, etc.

"The plant is under the direction of the federal authorities, and their intention is not only to use it for disinfection of baggage, etc., coming into Mexico, but also going out of Mexico, when necessary. The disinfection of persons and baggage from Vera Cruz, and other yellow fever points, for example, will be done by the Mexican authorities under the inspection of the Texas Quarantine Officer, who can then, if he sees proper, give a permit to pass through Texas.

"The plan seems to me to be a very good one, theoretically.

Whether it will work without friction, depends upon whether Mexican officials really disinfect or not.

"They will have, of course, better facilities for finding out where passengers are from, and could, therefore, aid us very materially in making our quarantine a perfect one."

